# Procaine Induces Epigenetic Changes in HCT116 Colon Cancer Cells

**DOI:** 10.1155/2016/8348450

**Published:** 2016-10-24

**Authors:** Hussein Sabit, Mariam B. Samy, Osama A. M. Said, Mokhtar M. El-Zawahri

**Affiliations:** ^1^College of Biotechnology, Misr University for Science and Technology, Giza, Egypt; ^2^Center of Research and Development, Misr University for Science and Technology, Giza, Egypt

## Abstract

Colon cancer is the third most commonly diagnosed cancer in the world, and it is the major cause of morbidity and mortality throughout the world. The present study aimed at treating colon cancer cell line (HCT116) with different chemotherapeutic drug/drug combinations (procaine, vorinostat “SAHA,” sodium phenylbutyrate, erlotinib, and carboplatin). Two different final concentrations were applied: 3 *μ*M and 5 *μ*M. Trypan blue test was performed to assess the viability of the cell before and after being treated with the drugs. The data obtained showed that there was a significant decrease in the viability of cells after applying the chemotherapeutic drugs/drug combinations. Also, DNA fragmentation assay was carried out to study the effect of these drugs on the activation of apoptosis-mediated DNA degradation process. The results indicated that all the drugs/drug combinations had a severe effect on inducing DNA fragmentation. Global DNA methylation quantification was performed to identify the role of these drugs individually or in combination in hypo- or hypermethylating the CpG dinucleotide all over the genome of the HCT116 colon cancer cell line. Data obtained indicated that different combinations had different effects in reducing or increasing the level of methylation, which might indicate the effectiveness of combining drugs in treating colon cancer cells.

## 1. Introduction

Cancer, the uncontrolled cell growth, is one of the most fatal diseases worldwide [1]. One of the most widespread and common types of cancer is colorectal cancer (CRC), which represents the third most common cancer after lung and breast cancers, and it is considered the second most common cause of cancer death [2–4].

It is well known that epigenetic alterations, particularly in the disease-related genes, are associated with various disorders including many cancer types [5]. Colorectal carcinoma is one of those diseases in which epigenetic inactivation of multiple tumor suppressor genes plays a crucial role in the tumorigenesis process [6].

The role of DNA methylation in the organization of the cancer epigenetic profile is still unclear. However, several studies have been conducted on HCT116 colon cancer cells to elucidate the landscape of DNA methylation [7, 8].

Chemotherapy is considered one of the effective therapeutic ways to control several types of cancer, although the standard chemotherapy plans often have limited survival benefits due to its severe cellular toxicity and the inability to target only the malignant cells [9]. Unfortunately, this off-targeting highlights the main concern of using chemotherapy [10].

Several literatures have focused on the application of a combination of chemotherapeutic drugs to treat cancer [11–14].

Procaine is one of the conventional chemotherapeutic agents, which was used also as a local anesthetic drug in surgeries. It showed an epigenetic mode of action, as a demethylating agent for the hypermethylated CpG island of DNA, and hence, it became one of the choices in treating different types of cancers [15].

Here, the main aim of the present study was to identify the role of procaine (as a representative of DNMT inhibitor drugs) combined with other chemotherapeutic drugs such as carboplatin (as a representative of DNMT inhibitor drugs), erlotinib, sodium phenylbutyrate, and vorinostat (as representatives of HDAC inhibitor drugs) in demethylating the whole genome of the HCT116 colon cancer cells.

## 2. Materials and Methods

### 2.1. Cell Line Maintenance

Colon cancer cell line (HCT116) was purchased from the Holding Company for Vaccines and Biological Products (VACSERA), Cairo, Egypt. Cells were cultured in RPMI-1640 media supplemented with 10% fetal bovine serum (FBS) and 1% antibiotic mix (ampicillin/streptomycin). Cells were maintained under the normal laboratory conditions, that is, 5% CO_2_ at 37°C.

### 2.2. Cell Viability Test

Trypan blue is a vital stain that is used to selectively color dead cells blue, while leaving live cells with intact cell membranes not colored. It was conducted to assess the number of cells before and after treatment with the chemotherapeutic drugs. The test is straightforward. Briefly, cell suspension was diluted with equal volume (1 : 1) of the dye and left for 3 min and then loaded to the hemocytometer slide. Cells were counted under inverted microscope as the bright cells were considered viable while the blue ones were considered dead. The total number of viable cells was calculated using the following equations:

(1) The total number of viable cells=Average number of viable cells×dilution factor×104.

### 2.3. Chemotherapy Drugs

Five chemotherapeutic drugs, procaine, carboplatin, vorinostat, sodium phenylbutyrate, and erlotinib, were used. All the drugs were purchased from Santa Cruz Biotechnology, USA. These drugs represent two groups of chemotherapeutic drugs: HDAC inhibitor and DNMT inhibitor.

### 2.4. Drug Preparation and Application

Five micrograms of each drug was dissolved into 5 mL of injection water to prepare the stock solutions 1 *μ*g/mL. Final concentration of 3 *μ*M and 5 *μ*M was prepared and applied to the HCT116 cells cultured in a 6-well plate. Two plates were used, one for each concentration. The 6-well plate layouts are presented in Tables 1 and 2.

### 2.5. Cell Harvesting

Cells were harvested for the downstream analysis after 3 days of incubation with drugs. Briefly, old media were decanted and the cells were trypsinized for 2 min and then collected* via* low speed centrifugation (200 rpm for 10 min). Cell viability was assessed also after treatment.

### 2.6. DNA Extraction

Total DNA from all samples was extracted using G-Spin™ Total DNA Extraction Kit (Boca Scientific, USA). The extracted DNA was used in both DNA degradation assay and methylation quantification.

### 2.7. DNA Degradation Assay

The extracted DNA from all samples was subjected to electrophoresis by loading a suitable volume on 1.2% agarose gel. Initial voltage (15 volts) for 5 min was applied and then the run was continued at 120 volts for 30 min. Gels were visualized and photographed under UV transilluminator after being stained with ethidium bromide.

### 2.8. Quantification of DNA Methylation

The extracted DNA from each sample was used to quantify the global DNA methylation using Global DNA Methylation ELISA Kit (Cell Biolabs Inc., USA). Briefly, a standard curve was initially generated and the kit's instruction was followed. The OD was read at 450 nm using plate reader.

### 2.9. Statistical Analysis

All statistical analyses were performed with SAS statistical software (SAS Institute, Cary, NC). Data were analyzed using the 2-factor repeated measures with interaction of analysis of variance (ANOVA) general linear models (GLM) procedure. Values were given as mean ± SD and differences among means were separated by Duncan's multiple range tests. A *P* value of 0.01 was considered significant. Correlation between variables was performed using Pearson correlation coefficient analysis.

## 3. Results

### 3.1. Cell Viability after Treatment

**Figure 1:**
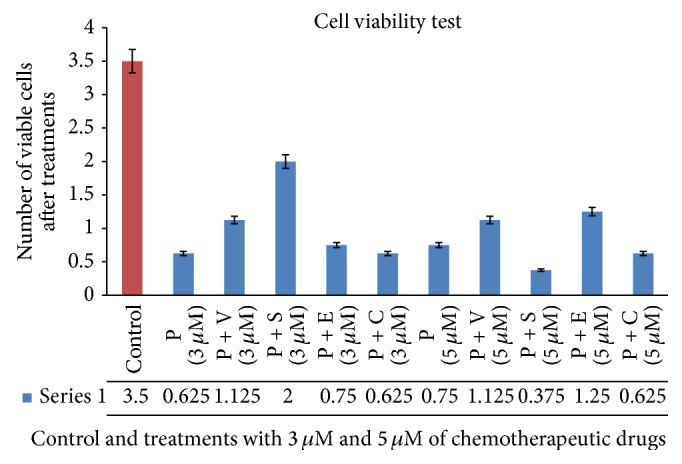
The effect of different drug combinations/concentration on the viability of HCT116 colon cancer cells compared to control. P: procaine, V: vorinostat, S: sodium phenylbutyrate, E: erlotinib, and C: carboplatin. Numbers are multiplied with 10^5^.

**Figure 2:**
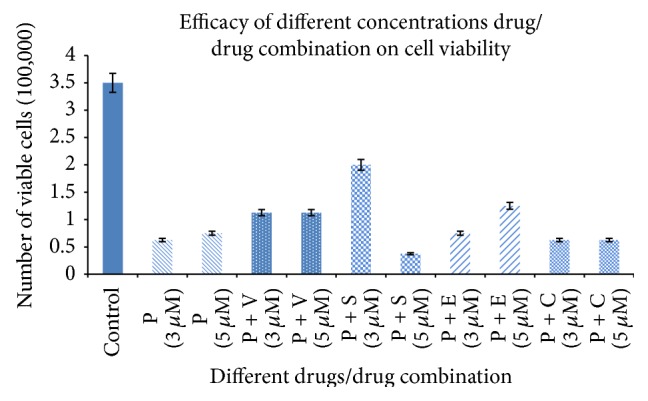
Drug combinations/concentration efficacy on the viability of HCT116 colon cancer cells, organized in drug-wise. P: procaine, V: vorinostat, S: sodium phenylbutyrate, E: erlotinib, and C: carboplatin. Numbers are multiplied with 10^5^.

Trypan blue assay was performed to assess the cell viability after treatment with the chemotherapeutic drug/drugs combinations as it can stain the dead cells while leaving the viable cells unstained. Results obtained showed that there was a significant decrease in the cell viability after being treated with different concentrations/combinations of the chemotherapeutics under study (Figures 1 and 2 and Table 3) (*P* < 0.01). Meanwhile, the most effective drug/combination was procaine combined with sodium phenylbutyrate at a concentration of 5 *μ*M (37,500 viable cells). While the same combination had less effect on malignant cell viability (200,000 viable cells) when applied at a lower concentration (3 *μ*M). This might indicate the efficacy of the higher doses of the combined chemotherapeutic drugs, despite the profile obtained with procaine combined with erlotinib as the low concentration of this combination gave better efficacy.  Figure 2 highlights a noticeable pattern as there was no significant variation of the number of viable cells when changing the drug/drug combination concentration. This pattern has been shown when using procaine, procaine combined with vorinostat, and procaine combined with carboplatin.

### 3.2. DNA Degradation Assay

**Figure 3:**
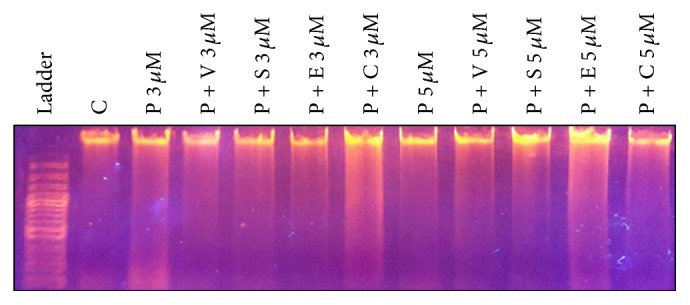
DNA degradation assay of treated and nontreated HCT116 colon cancer cells. P: procaine, V: vorinostat, S: sodium phenylbutyrate, E: erlotinib, and C: carboplatin.

Chemotherapeutic drugs' effect can be studied by studying DNA degradation [16]. In the present study, DNA fragmentation was assessed in the HCT116 colon cancer cells after being treated with different drugs/drug combinations. Data obtained (Figure 3) indicated the severe damage in cellular DNA of all cells regardless of the drug/drug combination. However, the most effective drug/drug combination in inducing DNA fragmentation was procaine alone (3 *μ*M) followed by procaine combined with both carboplatin (3 *μ*M) and erlotinib (5 *μ*M).

### 3.3. Quantification of DNA Methylation

**Figure 4:**
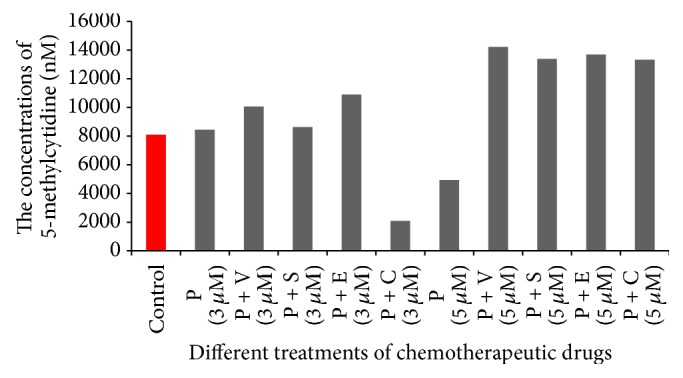
The concentration of 5-methylcytidine in all cells after being treated with different chemotherapeutic drugs compared to control. P: procaine, V: vorinostat, S: sodium phenylbutyrate, E: erlotinib, and C: carboplatin.

**Figure 5:**
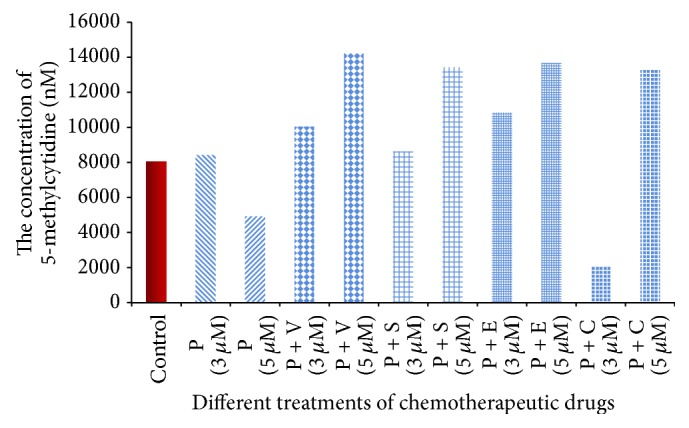
The concentration of 5-methylcytidine of treated and nontreated HCT116 colon cancer cells, organized drug-wise. P: procaine, V: vorinostat, S: sodium phenylbutyrate, E: erlotinib, and C: carboplatin.

In colon cancer, epigenetic changes, such as promoter CpG island hypermethylation, resulted from the expression of DNMT. Promoter methylation occurs more frequently than genetic mutations [17, 18], so quantification of the global methylome in colon cancer cells might provide insights about the epigenetic changes, particularly after being treated with chemotherapy. In the present study the global methylation pattern was identified to evaluate the role of procaine associated with other drugs in changing the methylation profile in colon cancer cells (Figures 4 and 5 and Table 4). Results obtained indicated that procaine alone at higher concentration (5 *μ*M) and procaine combined with carboplatin in the lower concentration (3 *μ*M) were the most efficient drug/drug combination in demethylating the whole genome of colon cancer cells compared to control (*P* < 0.01). However, the same combination in the higher concentration (5 *μ*M) was able to hypermethylate the whole genome of the cells compared to control. Meanwhile procaine combined with the other drugs, that is, vorinostat (HDAC inhibitor), sodium phenylbutyrate (HDAC inhibitor), and erlotinib (HDAC inhibitor), in higher concentration (5 *μ*M) was also able to increase the methylation level significantly compared to control. This might indicate that combining procaine with these drugs might antagonize its DNMT inhibitory effect and lead to promotion of the cell proliferation and hence increase the total methylation amount.

However, data showed (Figure 5) a general trend of a correlation between the methylation level and the drug doses. The lower doses (3 *μ*M) of procaine combined with vorinostat, sodium phenylbutyrate, erlotinib, and carboplatin were more effective in reducing the methylation level of the HCT116 colon cancer cells compared to the high doses (5 *μ*M) of the same combinations. The only exception appears when procaine was used solely as the high dose was more effective than the low dose in reducing the methylation level. The present study, therefore, indicates the effectiveness of using lower doses of the specified chemotherapy.

## 4. Discussion

### 4.1. Cell Viability

One of the most direct tools to identify the effect of any treatment on malignant cells is assessing the cell viability [19]. The significant decrease of the number of viable cells compared to control could be attributed to the activation of extrinsic apoptotic pathway through which the procaine combined with sodium phenylbutyrate demethylated some apoptosis related gene, which, in turn, activated the apoptosis machinery and then cell death [20, 21].

Several studies have indicated the efficacy of chemotherapeutic drugs/drug combinations in reducing malignant cell counts [22, 23]. Procaine was the main drug applied to the HCT116 colon cancer cells. When it was applied solely (either in 3 or 5 *μ*M), a significant reduction in the cell count was obtained. This might indicate that procaine was able to enforce the cells to commit apoptosis. Other research groups have indicated the same profile [24–26]. A reduction in the cell count has also been obtained when procaine was combined with vorinostat in both concentrations, and similar profiles were obtained by other research groups [25, 27]. Meanwhile, in this combination, the dose has no significant effect, which might indicate the differences in the role of both procaine and vorinostat. When combined with sodium phenylbutyrate, procaine in both concentrations exhibited an ability to significantly reduce the cell count which was lower than using procaine solely. This might highlight the antagonistic effect of sodium phenylbutyrate when combined with procaine, in which the former drug inhibited procaine from performing its action [28–31].

However, when procaine was combined with either erlotinib or carboplatin, a significant reduction in the cells count was obtained. This data might also show that erlotinib and carboplatin have also a differed mode of action compared to procaine. Several studies have identified similar outcomes [32–35].

### 4.2. DNA Degradation Assay

Several mechanisms could explain the DNA degradation in cells treated with chemotherapeutic drugs. One of these mechanisms postulates that demethylating agent such as procaine could activate tumor suppressor gene(s), which, in turn, enforce the cells to commit apoptosis [36, 37]. Other mechanisms suggest the activation of caspase-activated DNase (CAD), which degrades DNA after a cascade of activation processes [38]. In the present investigation all drug combinations applied to HCT116 colon cancer cells have resulted in a degradation pattern that might indicate the occurrence of drugs-induced apoptosis [27, 39, 40].

### 4.3. Quantification of DNA Methylation

DNA methylation quantification is considered one of the most widespread tools to assess the nonsequence dependent gene regulation [41]. In the present study, global methylation was quantified in the treated and untreated HCT116 colon cancer cells. Results obtained indicated that procaine applied solely (3 *μ*M) has increased the methylation level compared to the control untreated cells, while the same compound in the higher dose (5 *μ*M) has resulted in decreased methylation level compared to control. This data might indicate that procaine applied solely activated DNMT and hence increased the methylation level. This data was also noticed by several research groups [42–44]. Meanwhile, when procaine was combined with vorinostat or sodium phenylbutyrate or erlotinib, a hypermethylation pattern was obtained in the lower concentration (3 *μ*M), while the higher concentration (5 *μ*M) gave a significant increase in the methylation level compared either to control or to the lower concentration. This data might indicate that combining procaine with either vorinostat or sodium phenylbutyrate or erlotinib has a synergistic effect on activating DNMT. This profile was observed in several previously published researches [17, 39, 45, 46]. However, when procaine was combined with carboplatin, the lower dose (3 *μ*M) has resulted in hypomethylating the whole genome of HCT116 colon cancer cells, and this might indicate the activity of this combination in inactivating DNMT [32, 42], while the higher dose has resulted in hypermethylation of the whole genome compared to control [47, 48].

## 5. Conclusion

Colon cancer is one of the leading causes of death worldwide. Therefore, the seeking for effective chemotherapeutic drugs or drug combinations is still a big demand. In the present study, HCT116 colon cancer cell line was treated with two concentrations (3 *μ*M and 5 *μ*M) of procaine solely or combined with other drugs aiming to control the disease. Global DNA methylation and the cell viability were assessed. Data showed that using procaine combined with carboplatin in low dose (3 *μ*M) was the most effective treatment that was capable of reducing the level of global methylation. On the other hand, data indicated that applying higher doses of the drugs under study has resulted in promoting the cell proliferation and hence the methylation amount. Meanwhile, using the lower doses of the specified drugs was more effective in controlling colon cancer cells. However, further analysis is required to elucidate the mechanism by which the higher doses promoted the proliferation of HCT116 colon cancer cells.

## Figures and Tables

**Table 1:** The 6-well plate layout for the concentration of 3 *µ*M.

Control	2 *µ*L P + 2498 *µ*L cell suspension^*∗*^	2 *µ*L P + 2 *µ*L V + 2496 *µ*L cell suspension^*∗*^

2 *µ*L P + 2 *µ*L S + 2496 *µ*L cell suspension^*∗*^	2 *µ*L P + 3 *µ*L E + 2495 *µ*L cell suspension^*∗*^	2 *µ*L P + 5 *µ*L C + 2493 *µ*L cell suspension^*∗*^

P: procaine, V: vorinostat, S: sodium phenylbutyrate, E: erlotinib, and C: carboplatin.

^*∗*^Cell  suspension was 10^6^ cells per mL.

**Table 2:** The 6-well plate layout for the concentration of 5 *μ*M.

Control	3 *μ*L P + 2497 *μ*L cell suspension^*∗*^	3 *μ*L P + 4 *μ*L V + 2493 *μ*L cell suspension^*∗*^

3 *μ*L P + 3 *μ*L S + 2494 *μ*L cell suspension^*∗*^	3 *μ*L P + 5 *μ*L E +2492 *μ*L cell suspension^*∗*^	3 *μ*L P + 8 *μ*L C + 2489 *μ*L cell suspension^*∗*^

P: procaine, V: vorinostat, S: sodium phenylbutyrate, E: erlotinib, and C: carboplatin.

^*∗*^Cell  suspension was 10^6^ cells per mL.

**Table 3:** The mean values of Duncan's multiple range test for cell viability of control and treated cells.

Treat./Conc.	*N*	Mean	Duncan grouping				
Control	4	350875	A				
P + S/3 *μ*M	4	200000		B			
P + E/5 *μ*M	4	124875			C		
P + V/3 *μ*M	4	112500			C	D	
P + V/5 *μ*M	4	112500			C	D	
P + E/3 *μ*M	4	75000			C	D	E
P/5 *μ*M	4	75000			C	D	E
P + C/3 *μ*M	4	62575				D	E
P/3 *μ*M	4	62500				D	E
P + C/5 *μ*M	4	62500				D	E
P + S/5 *μ*M	4	37500					E

P: procaine, V: vorinostat, S: sodium phenylbutyrate, E: erlotinib, and C: carboplatin.

**Table 4:** Mean values of Duncan's multiple range test for quantification of global DNA methylation.

Treat./Conc.	*N*	Mean	Duncan grouping		
P + V/5 *μ*M	4	14100	A		
P + E/5 *μ*M	4	14000	A		
P + S/5 *μ*M	4	13900	A		
P + C/5 *μ*M	4	13900	A		
P + E/3 *μ*M	4	10400	A	B	
P + V/3 *μ*M	4	10000	A	B	
P + S/3 *μ*M	4	8300		B	
P/3 *μ*M	4	8200		B	
Control	4	8000		B	
P/5 *μ*M	4	5100		B	C
P + C/3 *μ*M	4	2200			C

P: procaine, V: vorinostat, S: sodium phenylbutyrate, E: erlotinib, and C: carboplatin.

## Additional Points

*Study Limitation*. Here in the present study, we assessed the role of different chemotherapeutic drugs in altering the global methylation pattern of HCT116 colon cancer cells. Despite the obtained data, this study needs more conformational investigation to deeply assess the mode of action of each combination applied. Meanwhile, the drug combinations used in this study are not intended for clinical uses.

## References

[1] López-Gómez M., Malmierca E., de Górgolas M., Casado E. (2013). Cancer in developing countries: the next most preventable pandemic. The global problem of cancer. *Critical Reviews in Oncology/Hematology*.

[2] Esposito K., Chiodini P., Capuano A. (2012). Colorectal cancer association with metabolic syndrome and its components: a systematic review with meta-analysis. *Endocrine*.

[3] Gado A., Ebeid B., Abdelmohsen A., Axon A. (2014). Colorectal cancer in Egypt is commoner in young people: is this cause for alarm?. *Alexandria Journal of Medicine*.

[4] Zheng W., Zhao L., Wang G. (2016). Promoter methylation and expression of RASSF1A genes as predictors of disease progression in colorectal cancer. *International Journal of Clinical and Experimental Medicine*.

[5] Cacabelos R. (2015). Epigenetic biomarkers in cancer. *Clinical & Medical Biochemistry*.

[6] Sakai E., Nakajima A., Kaneda A. (2014). Accumulation of aberrant DNA methylation during colorectal cancer development. *World Journal of Gastroenterology*.

[7] Michailidi C., Theocharis S., Tsourouflis G. (2015). Expression and promoter methylation status of hMLH1, MGMT, APC, and CDH1 genes in patients with colon adenocarcinoma. *Experimental Biology and Medicine*.

[8] Jones P. A. How DNA methylation organizes the cancer epigenome.

[9] Yin S., Wei W., Jian F., Yang N. (2013). Therapeutic applications of herbal medicines for cancer patients. *Evidence-Based Complementary and Alternative Medicine*.

[10] Kumar S. P., Sisodia V. (2013). Chemotherapy-induced or chemotherapy-associated? Does physical therapy play a role in prevention and/or management of peripheral neurotoxicity and neuropathy?. *Indian Journal of Palliative Care*.

[11] Durant J., Clevenbergh P., Halfon P. (1999). Drug-resistance genotyping in HIV-1 therapy: the VIRADAPT randomised controlled trial. *Lancet*.

[12] da Silva P. E. A., Palomino J. C. (2011). Molecular basis and mechanisms of drug resistance in *Mycobacterium tuberculosis*: classical and new drugs. *Journal of Antimicrobial Chemotherapy*.

[13] Borst P., Jonkers J., Rottenberg S. (2007). What makes tumors multidrug resistant?. *Cell Cycle*.

[14] Xie L., Xie L., Kinnings S. L., Bourne P. E. (2012). Novel computational approaches to polypharmacology as a means to define responses to individual drugs. *Annual Review of Pharmacology and Toxicology*.

[15] Dhivya S., Khandelwal N., Abraham S. K., Premkumar K. (2016). Impact of anthocyanidins on mitoxantrone-induced cytotoxicity and genotoxicity: an in vitro and in vivo analysis. *Integrative Cancer Therapies*.

[16] Saadat Y. R., Saeidi N., Vahed S. Z., Barzegari A., Barar J. (2015). An update to DNA ladder assay for apoptosis detection. *BioImpacts*.

[17] Flis S., Gnyszka A., Flis K. (2014). DNA methyltransferase inhibitors improve the effect of chemotherapeutic agents in SW48 and HT-29 colorectal cancer cells. *PLoS ONE*.

[18] Ng J. M.-K., Yu J. (2015). Promoter hypermethylation of tumour suppressor genes as potential biomarkers in colorectal cancer. *International Journal of Molecular Sciences*.

[19] Rivaa G., Baronchellia S., Paolettaa L. (2014). In vitro anticancer drug test: a new method emerges from the model of glioma stem cells. *Toxicology Reports*.

[20] Lee M. H., Yang J. Y., Cho Y. (2016). Menadione induces apoptosis in a gastric cancer cell line mediated by down-regulation of X-linked inhibitor of apoptosis. *International Journal of Clinical and Experimental Medicine*.

[21] Lin J., Yao H.-J., Li R.-Y. (2016). Bakuchiol inhibits cell proliferation and induces apoptosis and cell cycle arrest in SGC-7901 human gastric cancer cells. *Biomedical Research*.

[22] Andre N., Schmiegel W. (2005). Chemoradiotherapy for colorectal cancer. *Gut*.

[23] Nautiyal J., Kanwar S. S., Yu Y., Majumdar A. P. N. (2011). Combination of dasatinib and curcumin eliminates chemo-resistant colon cancer cells. *Journal of Molecular Signaling*.

[24] Villar-Garea A., Fraga M. F., Espada J., Esteller M. (2003). Procaine is a DNA-demethylating agent with growth-inhibitory effects in human cancer cells. *Cancer Research*.

[25] Brueckner B., Boy R. G., Siedlecki P. (2005). Epigenetic reactivation of tumor suppressor genes by a novel small-molecule inhibitor of human DNA methyltransferases. *Cancer Research*.

[26] Stresemann C., Brueckner B., Musch T., Stopper H., Lyko F. (2006). Functional diversity of DNA methyltransferase inhibitors in human cancer cell lines. *Cancer Research*.

[27] Sachan M., Kaur M. (2015). Epigenetic modifications: therapeutic potential in cancer. *Brazilian Archives of Biology and Technology*.

[28] Rodríguez-Paredes M., Esteller M. (2011). Cancer epigenetics reaches mainstream oncology. *Nature Medicine*.

[29] Walia Y. K., Sharma V. (2013). Role of HDACs and DNMTs in cancer therapy: a review. *Asian Journal of Advanced Basic Sciences*.

[30] Ushijima M., Ogata Y., Tsuda H., Akagi Y., Matono K., Shirouzu K. (2014). Demethylation effect of the antineoplaston AS2-1 on genes in colon cancer cells. *Oncology Reports*.

[31] Burzynski S. R., Janicki T. J., Burzynski G. S., Brookman S. (2014). Preliminary findings on the use of targeted therapy in combination with sodium phenylbutyrate in colorectal cancer after failure of second-line therapy—a potential strategy for improved survival. *Journal of Cancer Therapy*.

[32] Amatori S., Bagaloni I., Donati B., Fanelli M. (2010). DNA demethylating antineoplastic strategies: a comparative point of view. *Genes and Cancer*.

[33] Kim M. S., Lee J., Sidransky D. (2010). DNA methylation markers in colorectal cancer. *Cancer and Metastasis Reviews*.

[34] Townsley C. A., Major P., Siu L. L. (2006). Phase II study of erlotinib (OSI-774) in patients with metastatic colorectal cancer. *British Journal of Cancer*.

[35] Mitchell S. M., Ross J. P., Drew H. R. (2014). A panel of genes methylated with high frequency in colorectal cancer. *BMC Cancer*.

[36] Lai D., Visser-Grieve S., Yang X. (2012). Tumour suppressor genes in chemotherapeutic drug response. *Bioscience Reports*.

[37] Xu J.-H., Hu S.-L., Shen G.-D., Shen G. (2016). Tumor suppressor genes and their underlying interactions in paclitaxel resistance in cancer therapy. *Cancer Cell International*.

[38] Gao L., Huang K., Jiang D.-S. (2015). Novel role for caspase-activated DNase in the regulation of pathological cardiac hypertrophy. *Hypertension*.

[39] Nebbiosoa A., Carafaa V., Benedettia R., Altuccia L. (2012). Trials with ‘epigenetic’ drugs: an update. *Molecular Oncology*.

[40] Witta S. (2012). Histone deacetylase inhibitors in non-small-cell lung cancer. *Journal of Thoracic Oncology*.

[41] Fouse S. D., Nagarajan R. P., Costello J. F. (2010). Genome-scale DNA methylation analysis. *Epigenomics*.

[42] Lyko F., Brown R. (2005). DNA methyltransferase inhibitors and the development of epigenetic cancer therapies. *Journal of the National Cancer Institute*.

[43] Johnson A. A., Akman K., Calimport S. R. G., Wuttke D., Stolzing A., de Magalhães J. P. (2012). The role of DNA methylation in aging, rejuvenation, and age-related disease. *Rejuvenation Research*.

[44] Delpu Y., Cordelier P., Cho W. C., Torrisani J. (2013). DNA methylation and cancer diagnosis. *International Journal of Molecular Sciences*.

[45] Kang K. A., Piao M. J., Kim K. C. (2014). Epigenetic modification of Nrf2 in 5-fluorouracil-resistant colon cancer cells: involvement of TET-dependent DNA demethylation. *Cell Death and Disease*.

[46] Wongtrakoongate P. (2015). Epigenetic therapy of cancer stem and progenitor cells by targeting DNA methylation machineries. *World Journal of Stem Cells*.

[47] Ren J., Singh B. N., Huang Q. (2011). DNA hypermethylation as a chemotherapy target. *Cellular Signalling*.

[48] Subramaniam D., Thombre R., Dhar A., Anant S. (2014). DNA methyltransferases: a novel target for prevention and therapy. *Frontiers in Oncology*.

